# Supplementary material for: Monitoring of mitochondrial oxygen tension in the operating theatre: An observational study with the novel COMET® monitor
Source: PLoS One. 2023 Feb 9;18(2):e0278561. doi: 10.1371/journal.pone.0278561 (PMC9910761; doi:10.1371/journal.pone.0278561)
Supplement: S1 File — (DOCX) [file pone.0278561.s001.docx]

Supplementary file 1. Illustrative cases demonstrating the behaviour of mitoPO_2_ in hemodynamically unstable conditions

The patients presented in the cases below have been included in the study ‘Noninvasive measurement of mitochondrial oxygen tension (mitoPO2) compared to continuous hemoglobin-monitoring during major surgery (NIMMOT)’. This study has been approved by the Institutional Review Board (IRB) at the Erasmus Medical Center CCMO-register (NL 63158. 078.17). All study procedures were performed in accordance with the relevant guidelines and regulations.

**Cases**

*Case A: pancreaticoduodenectomy with significant blood loss*

A 61-year-old ASA II male suspected of having a cholangiocarcinoma underwent an open pancreaticoduodenectomy according to the WHIPPLE procedure. The patient had a minimal medical history having a cholecystitis for which several ERCP procedures and a laparoscopic cholecystectomy had been performed. Preoperative physical, abdominal, respiratory and cardiac examination were unremarkable and the patient did not take any home medication. The results of the preoperative blood test are summarized in table S1.

| **Table S1. Preoperative laboratory assessment** | | | |
| --- | --- | --- | --- |
| **Laboratory test (unit)** | **Case A** | **Case B** | **Ref. value** |
| Glucose (mg/dL) | 185.4 | 102.6 | 72.0-109.8 |
| Creatinine (mg/dL) | 0.78 | 0.83 | 0.74-1.30 |
| Hemoglobin (g/dL) | 16.3 | 14.2 | 13.9-16.9 |
| Platelets (cells/mm^3^) | 291.000 | 245.000 | 150.000-370.000 |
| Leukocytes (cells/mm^3^) | 6.6000 | 3.5000 | 3.5000-10.000 |

An epidural catheter was inserted before the induction of anesthesia and epidural analgesia with ropivacaine/sufentanil was given prior to surgery. For induction of anesthesia, an IV bolus of 250 mg propofol, 50 mg rocuronium and infusion of remifentanil 9 mcg/kg/h was used. Anesthesia was maintained by continuous infusion of propofol 8 mg/kg/h and remifentanil 8 mcg/kg/h. Directly after induction, continuous infusion of noradrenalin (0.05-0.19 mcg/kg/min) was necessary to maintain an adequate blood pressure. On top of standard perioperative monitoring consisting of invasive blood pressure measurements, peripheral oxygen saturation, electrocardiography, and temperature measurements, COMET^®^ was used for the mitoPO_2_ measurements, and continuous total hemoglobin (SpHb) measurements were performed with a disposable Masimo Rainbow adult adhesive sensor (R2-25a Masimo Corporation, Irvine, USA). Because the tumor was located near the portal vein the procedure was complicated and throughout the operation the patient lost 3750 ml of blood (figure S1). Resuscitation of blood loss was performed using crystalloid (NaCl 0,9% and Sterofundin), colloid solution (Voluven 6%) and vasoactive medication. However, the need for intraoperative blood transfusion did not arrive and intraoperative serum lactate remained low. At the end of the surgery there was a positive fluid balance of 1390ml. During surgery, the mitoPO_2_ value gradually declined from 79 mmHg to 5 mmHg. This decrease in mitoPO_2_ was accompanied by a decline in hemoglobin from 14.82 g/dL to 9.9 g/dL (determined by a blood gas analyzer). Continuous SpHb measurements were also executed and declined from 11.76 g/dL to 10.63 g/dL. Post-operatively the patient had increased lactate levels and eventually received a blood transfusion.


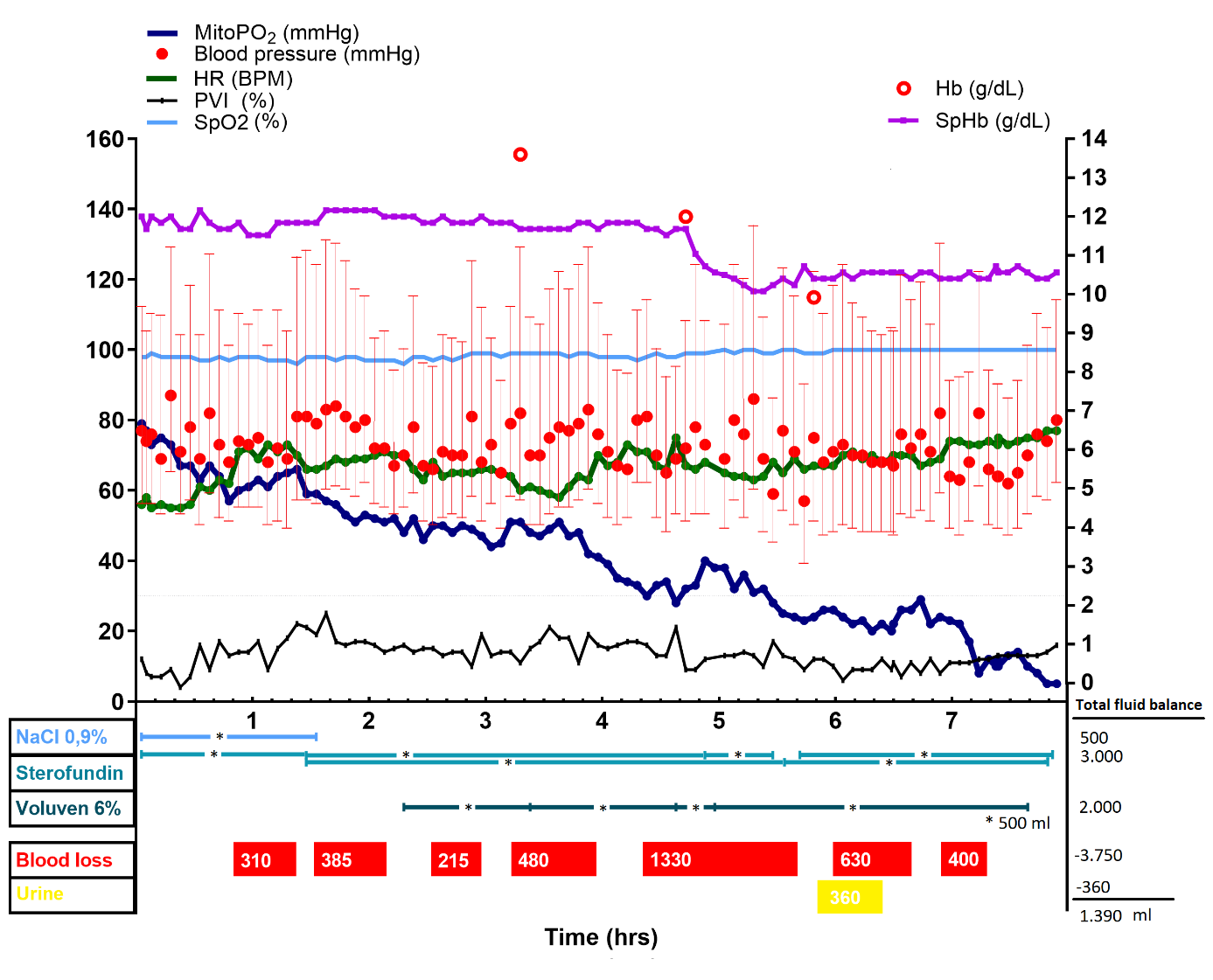


Figure S1: Representative case with significant amount of blood loss during pancreaticoduodenectomy. Blood pressure is presented in mean arterial pressure (dot) with the diastolic and systolic blood pressure represented by the bottom and top whiskers. MitoPO_2_; mitochondrial oxygen tension, Hb; hemoglobine, SpHb; peripheral hemoglobin, SpO2; peripheral oxygen saturation, PVI; pulse variation index, HR; heart rate

*Case B: Partial hepatectomy*

A 57-year-old ASA I female patient, underwent a partial hepatectomy because of a rapidly growing cyst. The patient’s relevant medical history includes a transient ischemic attack (TIA) in 2017. Her medication consisted of clopidogrel 75mg once a day, this was stopped 5 days prior to surgery. Preoperative abdominal, respiratory and cardiac examination were unremarkable. The blood test results are summarized in table 1.

An epidural catheter was inserted before induction of anesthesia and epidural analgesia with ropivacaine/sufentanil was given prior to surgery. For induction of anesthesia, an IV bolus of 140 mg propofol, 50 mg rocuronium and infusion of remifentanil 7 mcg/kg/h was used. Anesthesia was maintained by using sevoflurane and continuous infusion of remifentanil 5 mcg/kg/h and epidural analgesia. After induction, continuous infusion of noradrenalin, starting at 0.30 mcg/kg/min and gradually increasing to 0.60 mcg/kg/min, was necessary to maintain an adequate blood pressure. On top of the standard perioperative monitoring techniques namely, invasive blood pressure measurements, peripheral oxygen saturation, electrocardiography, temperature measurements and central venous pressure (CVP), the COMET^®^ was used for the mitoPO_2_ measurements, and continuous total hemoglobin (SpHb) measurements were performed with a disposable Masimo Rainbow adult adhesive sensor.

At the beginning of the surgery, 400 ml of blood was withdrawn to preserve a low CVP. To ensure a low CVP, fluid admission was kept to a minimum throughout the surgery. Only after the hepatectomy was completed, fluid resuscitation was commenced with an autologous blood transfusion followed by 1 liter of Gelofusine (figure S2). By the end of the surgery the fluid balance ended up being 900 ml positive. Besides the withdrawal of 400ml of blood which was later given as an autologous blood transfusion, a total of 200ml blood was lost during surgery. Under this controlled hypovolemia, the mitoPO_2_ dropped from 83 mmHg to values around 20 mmHg. Lactate levels rose from 9.0 mg/dL to 19.8 mg/dL. After the tumor was removed and volume status was restored to a normovolemic state, the mitoPO_2_ started to recover.


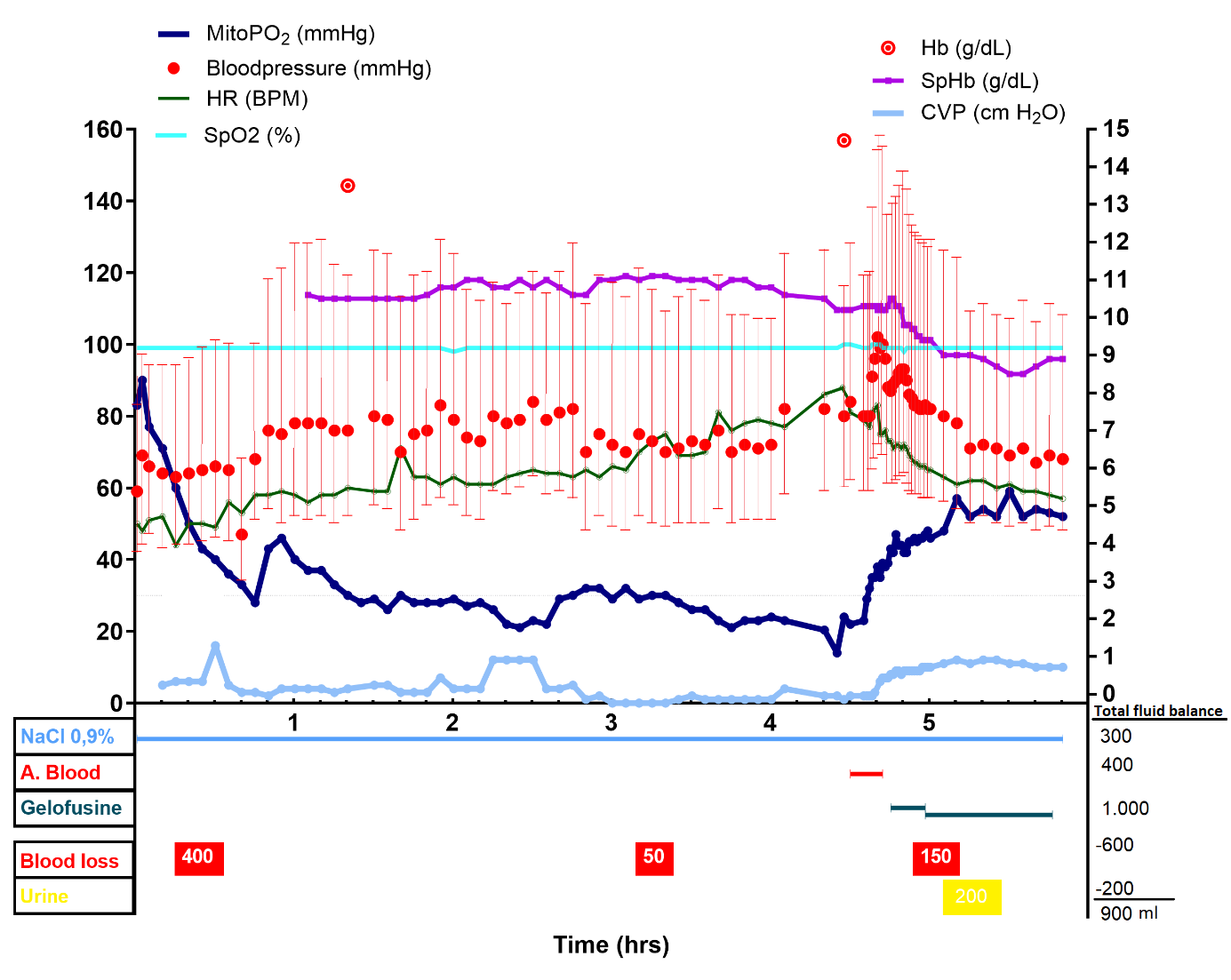


Figure S2: Representative case of a patient with controlled hypovolemia and recovery to normovolemia. Blood pressure is presented in mean arterial pressure (dot) with the diastolic and systolic blood pressure represented by the bottom and top whiskers. MitoPO_2_; mitochondrial oxygen tension, Hb; hemoglobine, SpHb; peripheral hemoglobin, SpO2; peripheral oxygen saturation, CVP; central venous pressure, HR; heart rate
