# Supplementary material for: Monitoring of mitochondrial oxygen tension in the operating theatre: An observational study with the novel COMET® monitor
Source: PLoS One. 2023 Feb 9;18(2):e0278561. doi: 10.1371/journal.pone.0278561 (PMC9910761; doi:10.1371/journal.pone.0278561)
Supplement: S2 File — (DOCX) [file pone.0278561.s002.docx]

Supplementary file 2. Results of LMM’s

**Table S1. Results of LMM mitoPO_2 ~_ skin temperature**

|  |  | **MitoPO_2_** |  |
| --- | --- | --- | --- |
| \| *Predictors* \| \| --- \| | *Estimates* | *CI* | *p* |
| (Intercept) | 108.03 | 79.59 – 136.48 | < 0.001 |
| Skin temperature | - 1.76 | - 2.55 - - 0.97 | < 0.001 |
| Time point | - 0.01 | - 0.01 – 0.00 | 0.076 |
| **Random effects** |  |  |  |
| σ2 | 36.32 |  |  |
| τ00 Timepoint | 10.86 |  |  |
| τ00 Subject | 176.07 |  |  |
| ICC | 0.84 |  |  |
| N Timepoint | 110 |  |  |
| N Subject | 20 |  |  |
| Observations | 748 |  |  |
| Marginal R­^2^ / Conditional R^2^ | 0.034 / 0.842 |  |  |

MitoPO­_2_; mitochondrial oxygen tension, CI; confidence interval, *p* ; p-value, σ2; variance of the residual, T00; random intercept variance, ICC; Intraclass Correlation Coefficient, N; number

**Table S2. Results of LMM mitoPO_2_ ~ skin temperature and covariates**

|  |  | **MitoPO_2_** |  |
| --- | --- | --- | --- |
| \| *Predictors* \| \| --- \| | *Estimates* | *CI* | *p* |
| (Intercept) | 374.23 | 263.60 – 484.87 | < 0.001 |
| Skin temperature | - 2.85 | - 3.86 – - 1.85 | < 0.001 |
| Time point | - 0.02 | - 0.03 – - 0.01 | < 0.001 |
| Flow | 0.07 | 0.01 – 0.13 | 0.015 |
| StO_2_ | 0.18 | 0.03 – 0.32 | 0.017 |
| Mean arterial pressure | 0.03 | - 0.05 – 0.12 | 0.416 |
| Heartrate | 0.02 | - 0.10 – 0.14 | 0.759 |
| SpO_2_ | - 2.60 | - 3.70 – - 1.51 | < 0.001 |
| FiO_2_ | 0.12 | - 0.01 – 0.25 | 0.072 |
| **Random effects** |  |  |  |
| σ2 | 38.36 |  |  |
| τ00 Time point | 7.53 |  |  |
| τ00 Subject | 203.73 |  |  |
| ICC | 0.85 |  |  |
| N Timepoint_new | 78 |  |  |
| N Subject | 14 |  |  |
| Observations | 483 |  |  |
| Marginal R­^2^ / Conditional R^2^ | 0.208 / 0.878 |  |  |

MitoPO­_2_; mitochondrial oxygen tension, CI; confidence interval, *p* ; p-value, N; number, σ2; variance of the residual, T00; random intercept variance, ICC; Intraclass Correlation Coefficient, StO_2_; capillary-venous oxygen  saturation, SpO_2_; peripheral oxygen saturation, FiO_2_; fraction of inspired oxygen
