# Supplementary material for: Monitoring of mitochondrial oxygen tension in the operating theatre: An observational study with the novel COMET® monitor
Source: PLoS One. 2023 Feb 9;18(2):e0278561. doi: 10.1371/journal.pone.0278561 (PMC9910761; doi:10.1371/journal.pone.0278561)
Supplement: Attachment — Submitted filename: PONE-D-21-32161_R-2022-07-02.pdf [file pone.0278561.s004.pdf]

## Title

Monitoring of mitochondrial oxygen tension in the operating theatre: an observational study with the novel COMET® monitor.

## Summary

The study aims to assess the feasibility and applicability of the COMET monitor in 20 patients undergoing neurosurgery under stable environmental conditions. Based on the PpIX-TSLT the COMET allows non-invasive, in-vivo measurements of mitochondrial oxygenation (mitoPO$_2$). Next to mitoPO$_2$ measurements, tissue oxygenation and local blood flow (Oxygen to see, O2C) and several other covariates were documented. In hemodynamically stable patients, the mitoPO$_2$ remained relatively stable. Nonetheless, especially in the first hour of surgery mitoPO2 tended to decrease in association with increasing probe temperature of the COMET.

## General impression

Overall, the authors present an interesting approach for mitochondrial $O_2$ monitoring in the operating theatre. Nonetheless, the authors should address some points before the manuscript is ready for publication.

## Abstract

**Minor:**

- L35: 60 ± 17 does ± refer to SD? Please clarify
- L36: Based on Figure 2 skin/probe temperature rose > consider adding with “increasing“ temperature
- L38: stable and unstable: in the current version of the manuscript the authors only present data on hemodynamically stable patients
- L40: Can the authors please comment on the exact meaning of “reliable”.
- L40-41: Please consider rephrasing the sentence.
- L41: Basically it was in neurosurgical patients.

## Introduction

**Major:**

- L74: „longitudinal observational pilot study“. I think this is not a classical longitudinal study.

**Minor:**

- IL61 [...] derived from muscle biopsies > consider adding a reference

# Methods

The methods part including trial design, setting and eligibility criteria as well as the principle of mitoP$O_2$ measurements is well written.

**Major:**

- L163-169: The sample size planning is not clear: "A sample size of 20 patients was calculated with an assumed mean difference of 12 mmHg and standard deviation of 18 mmHg, a type I error probability of 0.05 and a power of 80%." For what kind of test? In addition, there are no tests reported within the manuscript. Please clarify.

**Minor:**

- L175: one bracket too much

**General comments on the design:**

The authors state *"The goal of this study was to measure the mitoPO2 intraoperatively under stable hemodynamic conditions. Therefore, patients were excluded if there was hemodynamic instability during surgery namely, if blood transfusion was required, if the mean arterial pressure (MAP) decreased more than 25% from baseline or if high doses of vasopressors were needed (noradrenaline ≥ 0,10 mcg/kg/min or the equivalent dose of phenylephrine)"*. As outlined in the discussion (L323ff) mitoP$O_2$ was sensitive to blood loss. I think it would be very interesting to analyse the mitoP$O_2$ behaviour in patients with the above-mentioned changes instead of excluding them. Please comment.

The authors collected information on oxygenation with multiple instruments (O2C and other covariates). Why are no associative analysis (e.g. mixed model approach) reported? This would significantly enhance the message of the manuscript. Especially regarding the suspected association of mitoP$O_2$ and probe temperature.

Please consider the reporting of a statistic for stability/variability e.g. coefficient of variation.

# Results

**Minor:**

- L184: On average, the ALA patch was applied 17 ± 3.3 hours before surgery > this is rather long, please comment.
- Can the authors please provide at least mean signal quality with SD and/or 95%CI at the beginning and the end of the COMET measurements?

## Tables

- What is the rational of reporting dexamethasone use?

## Figures

- What does the N refer to? The number of patients for the obtained means and SDs? This should be stated in the figure legends.
- There might be confounding between the first data points with N=20 and last data points N=10, if individual patients start from different mitoP$O_2$ levels. Might the decrease of mitoP$O_2$ during the first hour go back to specific individuals? E.g. in Figure 3 (B) the mitoP$O_2$ seems rather stable.

## Discussion

L271: Please consider adding the reference.

L287-289: On average the mito$PO_2$ dropped from ~60 to ~40 mmHg. The probe temperature (and the skin temperature) increased from 34 to 35°C. The authors state that the decline in mito$PO_2$ most likely is not clinically relevant.

- What would be a clinical relevant change?
- The authors give a potential explanation for the association of skin temperature and mito$PO_2$. Is it reasonable that a rather small change in skin temperature is associated with a rather "large" change in mito$PO_2$?
- The association of probe/skin temperature could be tested using a mixed models approach. In addition, this approach could provide adjusted mito$PO_2$ values. If the negative trend over time in mito$PO_2$ is still present, other potential covariates should be discussed.
- Was there only an increase in skin temperature or also in body temperature?

L317: "and" value of [...]?

L323: Please consider adding the reference.

L348: What is the meaning of "evidence based monitoring of mito$PO_2$"?

L353: It should be stated that this was intraoperatively. Other studies reported COMET-measurements in healthy controls (hemodynamically stable).

L355: Only neurosurgical patients should be added.

***Additional topics, which could be addressed:***

Is the skin mito$PO_2$ a good surrogate parameter for the central or general oxygenation?

Only neurosurgical patients. What other body area would be feasible e.g. for cardiac surgery? Would the authors expect different mito$PO_2$ values in different body regions?

## General Comment

I see two options for this manuscript:

I: If the primary aim of the manuscript is to show the feasibility and applicability of COMET-measurements during (neuro-) surgery, some of the reported results could be shortened (e.g. O2C measurements). In addition, more results on signal quality changes, excluded data points etc. could be added.

II: The manuscript could additionally focus on associative analyses. The great advantage of the design is the parallel assessment of mito$PO_2$, O2C variables and other covariates. Nonetheless, I'm aware that this would switch the focus of the current manuscript and would be very time consuming.
