# Supplementary material for: Monitoring of mitochondrial oxygen tension in the operating theatre: An observational study with the novel COMET® monitor
Source: PLoS One. 2023 Feb 9;18(2):e0278561. doi: 10.1371/journal.pone.0278561 (PMC9910761; doi:10.1371/journal.pone.0278561)
Supplement: Attachment — Submitted filename: Rebuttal letter - plos one.docx [file pone.0278561.s003.docx]

Dear Editors,

Thank you for your time and expertise reviewing our manuscript. We have revised the manuscript based on the given comments, in detail:

1. Consider deleting the two cases and the related contents. *Author’s reply: we have deleted the cases and the related contents*
2. More information of intraoperative variables will be helpful, e.g., maintenance of anesthesia, amount of fluid supplement, and heart rate. *Author’s reply: we have added extra information of intraoperative variables, in particular maintenance of anesthesia, heart rate, oxygen saturation and inspired oxygen fraction*
3. LINE 104, the equation requires revision. *Author’s reply: We have revised the equation in the manuscript.*
4. Please clarify the clinical interpretation of mitoPO_2_. *Author’s reply: Since many factors are involved in maintaining an adequate cellular oxygenation, we suggest that perioperative hemodynamic monitoring, blood transfusion and fluid therapy strategies during complex surgeries should not be based on standard care intraoperative parameters alone. During these circumstances the addition of mitochondrial oxygenation and microvascular flow and saturation measurements can be essential to prevent cellular hypoxia and organ damage in order to improve long-term outcome. We have added this to the discussion section.*
5. Please clarify the “great value” for improving hemodynamic management, e.g., poor mitoPO2 masked by normal hemodynamic management under specific condition. *Author’s reply: In the current clinical practice hemodynamic optimization during major surgery remains challenging remains, partly due to the absence of an adequate end-point for tissue hypoxia. Therefore patients are often over or under resuscitated. Mitochondrial oxygenation and microvascular flow and saturation measurements can be essential to gain insight into the microcirculation and prevent cellular hypoxia and organ damage in order to improve long-term outcome. We have added this to the discussion section.*
6. Please list the limitations and strengths. *We have added the limitations and strengths*

*of the study to the discussion section.*

In addition to the revision of the manuscript we would like to add C.J. de Wijs to the authors list due to his contributions in reviewing and revising the manuscript and change the first authorship of F.A. Harms to co-first authorship with L.W.J.M. Streng considering her contributions to the measurements, data processing, writing and revising of the manuscript. All authors have given consent for this change.

Kind regards,

Floor Harms
