# Supplementary material for: Monitoring of mitochondrial oxygen tension in the operating theatre: An observational study with the novel COMET® monitor
Source: PLoS One. 2023 Feb 9;18(2):e0278561. doi: 10.1371/journal.pone.0278561 (PMC9910761; doi:10.1371/journal.pone.0278561)
Supplement: Attachment — Submitted filename: Answer to the reviewers_Harms_MitoPO2_OR.docx [file pone.0278561.s005.docx]

Answers to the comments of the reviewers on manuscript: Monitoring of mitochondrial oxygen tension in the operating theatre: an observational study with the novel COMET® monitor.

Reviewer #1: 
Comments:
1 .I would have expected more information in the introduction about mitoPO2 and the importance of its measurement. Comparison with other available methods and research of other groups in this field. A considerable amount of self-citation appears in the manuscript.
*Answer:* We have taken note of the reviewers suggestion and added an extra paragraph in the introduction on available different methods of measuring tissue PO_2_ and their limitations.

2. page6 line 104: equation is not well formatted.
*Answer:* The equation has been re-formatted.

3. page7 line 134: The use of ALA was indicated at least 5 hours before surgery. However, later in the text (page 10 line 176) the authors mention 17 hours before surgery. It would be useful to specify a time period when the measurement should and could be done, since ALA is metabolised later in the tissue.
*Answer:* This is a very useful suggestion. We have started our experiments with an application time of four hours, which is sufficient in optimal measuring circumstances. However, in clinical studies exact timing appeared to be an issue (e.g. due to delayed procedures and rescheduling) and we gained experience with longer application times. Additionally, longer application times appeared more convenient if we want to measure during surgeries, because four hours before the start of the surgery would mean application in the middle of the night in early surgeries. Up until now we have been able to obtain adequate measurements with application times ranging from 4 to 21 hours. However, based on experience we now do recommend at least 5 hours of application time when the measurements are performed in a clinical setting because of the better signal quality compared to 4 hours.

4) page 15 line 281: The authors mention previously published results, but there is no reference to this work.
*Answer:* We have added a reference after the above mentioned line.

5) The limitations and advantages of the experimental design should also be discussed.
*Answer:* The limitations and advantages have been added to the discussion.

6) page 16 line 312: mitoPO2 (lower index)
*Answer:* The 2 has been changed to subscript.

Reviewer #2:

Abstract

**Minor:**L35: 60 ± 17 does ± refer to SD? Please clarify

*Answer:* 60 ± 17 does refer to mean and standard deviation. We have also added this to the paragraph.

L36: Based on Figure 2 skin/probe temperature rose > consider adding with “increasing“ temperature

*Answer:* The word increasing has been added to the paragraph.

L38: stable and unstable: in the current version of the manuscript the authors only present data on hemodynamically stable patients

*Answer:* The word unstable has been removed from line 38.

L40: Can the authors please comment on the exact meaning of “reliable”.

*Answer:* The sentence has been rephrased.

L40-41: Please consider rephrasing the sentence.

*Answer:* The sentence has been rephrased

L41: Basically it was in neurosurgical patients.

*Answer:* ‘Major surgery’ has been changed to ‘measurements in the perioperative trajectory’.

Introduction

**Major:**

L74: „longitudinal observational pilot study“. I think this is not a classical longitudinal study.

*Answer:* All mentioning of a longitudinal study has been changed to “observational pilot study”.

**Minor:**

IL61 […] derived from muscle biopsies > consider adding a reference

*Answer:* The paragraph has been rephrased.

Methods

The methods part including trial design, setting and eligibility criteria as well as the principle of mitoPO2 measurements is well written.

**Major:**

L163-169: The sample size planning is not clear: “A sample size of 20 patients was calculated with an assumed mean difference of 12 mmHg and standard deviation of 18 mmHg, a type I error probability of 0.05 and a power of 80%.“ For what kind of test? In addition, there are no tests reported within the manuscript. Please clarify.

*Answer:* The sample size has been calculated for a t-test using G*power. We have added this in the paragraph.

**Minor:**

L175: one bracket too much

*Answer:* The bracket has been removed.

**General comments on the design:**

The authors state *“The goal of this study was to measure the mitoPO2 intraoperatively under stable hemodynamic conditions. Therefore, patients were excluded if there was hemodynamic instability during surgery namely, if blood transfusion was required, if the mean arterial pressure (MAP) decreased more than 25% from baseline or if high doses of vasopressors were needed (noradrenaline ≥ 0,10 mcg/kg/min or the equivalent dose of phenylephrine)”.* As outlined in the discussion (L323ff) mitoPO2 was sensitive to blood loss. I think it would be very interesting to analyse the mitoPO2 behaviour in patients with the above-mentioned changes instead of excluding them. Please comment.

*Answer:* The authors agree with the reviewer that measuring mitochondrial oxygenation in unstable hemodynamic conditions would be of interest. For example in patients with major blood loss. However, the primary aim of this study was to investigate the mitoPO_2_ in stable conditions, as it is the first time mitoPO_2_ was measured during surgery in adults. Seen as we do agree that unstable hemodynamic conditions are important as well, we are currently conducting measurements during major abdominal surgery in which major blood loss is expected. In a previous version of the manuscript, we had added two example cases from the previous mentioned population. However, these cases were removed after the editor’s comments. We have now added the two cases as a supplementary file.

The authors collected information on oxygenation with multiple instruments (O2C and other covariates). Why are no associative analysis (e.g. mixed model approach) reported? This would significantly enhance the message of the manuscript. Especially regarding the suspected association of mitoPO2 and probe temperature.

Please consider the reporting of a statistic for stability/variability e.g. coefficient of variation.

*Answer:* The authors agree with the reviewer that an associative analysis would be an important addition to the manuscript. Therefore, we have performed a mixed linear model analysis and added this to the manuscript. Furthermore, we have added a coefficient of variation in the text.

Results

**Minor:**

L184: On average, the ALA patch was applied 17 ± 3.3 hours before surgery > this is rather long, please comment.

*Answer*: The rather long application time is a result of practical reasons. Since the majority of the operations start at eight a.m., an application time of four hours would be in the middle of the night. In our other studies, including a cardiothoracic surgery population, COVID-19 patients and healthy volunteers, longer application times have proven to be effective. Therefore , we have chosen to place the plaster the afternoon or evening before the surgery [1].

We have added an explanation on application time in the discussion section.

Can the authors please provide at least mean signal quality with SD and/or 95%CI at the beginning and the end of the COMET measurements?

*Answer*: We have added mean signal quality with standard deviation at the beginning and end of the measurements to the results.

Additionally, we have added a paragraph on signal quality in the discussion. As after careful consideration, we have included the data of measurements with a signal quality under 20%. This is in contrast to the text in our study protocol and in the earlier version of this manuscript. We have had the possibility to analyse the data of the delayed fluorescence signal of the mitoPO_2_ data directly. When doing this, we learned that a signal quality of <20% as displayed by the COMET monitor does not necessarily reflect a signal to noise ratio of over 20. Therefore, with the current calculation of signal quality within the COMET, we see value in assessing the reliability of mitoPO­_2_ measurements based on the raw data, instead of excluding data points based on the % shown on the COMET monitor.

Tables

What is the rational of reporting dexamethasone use?

*Answer:* The rational for reporting dexamethasone use was the potential effect of dexamethasone on mitochondrial function. However, after consideration we have decided to leave it out of the table.

Figures

What does the N refer to? The number of patients for the obtained means and SDs? This should be stated in the figure legends.
*Answer:* The N stands for the number of patients for the obtained means and SDs, we have added this to the figure legends.

There might be confounding between the first data points with N=20 and last data points N=10, if individual patients start from different mitoPO2 levels. Might the decrease of mitoPO2 during the first hour go back to specific individuals? E.g. in Figure 3 (B) the mitoPO2 seems rather stable.

*Answer:* In the figure below we have plotted the mitoPO_2_ of every single patient separately. As can be seen, there is an overall decrease in all patients, not necessarily caused by specific individuals.

**Figure 1. MitoPO_2_ ­during neurosurgery. The lines represent the mitochondrial oxygenation (mitoPO_2_ ) of 20 individual patients.**

Discussion

L271: Please consider adding the reference.

*Answer:* The reference has been added.

L287-289: On average the mitoPO2 dropped from ~60 to ~40 mmHg. The probe temperature (and the skin temperature) increased from 34 to 35°C. The authors state that the decline in mitoPO2 most likely is not clinically relevant.

What would be a clinical relevant change?

*Answer:* In examples we have seen in ongoing trials, a mitoPO­_2_ below 20 mmHg seems to be clinically relevant. However, as it was not within the objectives of this study to investigate the significance of low mitoPO_2_ we cannot draw any definitive conclusions. Currently we are performing several studies (both in animal models as in humans) with the objective to study clinical relevant changes.

The authors give a potential explanation for the association of skin temperature and mitoPO2. Is it reasonable that a rather small change in skin temperature is associated with a rather “large” change in mitoPO2?

*Answer:* In order to further analyze the effect of skin temperature on mitoPO_2_ we have performed a linear mixed model analysis. In the model with only skin temperature as predictor variable, the estimate of the effect of skin temperature on mitoPO_2_ was -1.76. In the model with several other hemodynamic covariates, the effect of skin temperature on mitoPO_2_ was -2.76. However, we hypothesize that these changes are not large. As a it means that if a patient’s skin temperature is increased with 3 degrees, the mitoPO_2_ is lowered with 9 mmHg, which we do not deem clinically relevant. However such temperature swings are unlikely in procedures, unless the patients are actively cooled and re-heated.

The association of probe/skin temperature could be tested using a mixed models approach. In addition, this approach could provide adjusted mitoPO2 values. If the negative trend over time in mitoPO2 is still present, other potential covariates should be discussed.

*Answer:* In light of the reviewers suggestions, we have performed a mixed linear model including several other covariates and added the results to the manuscript.

Was there only an increase in skin temperature or also in body temperature?

*Answer:* Next to a change in skin temperature, a slight change in body temperature is also expected. The increase in skin temperature is presumably caused by the hot air blanket, which covers the patient during surgery. The blanket heats the skin with 43 degrees. Therefore the increase in skin temperature is believed to be higher than the expected increase in body temperature.

L317: “and” value of […]?

*Answer:* The letter ‘d’ has been added after ‘an’ in line 317.

L323: Please consider adding the reference.

*Answer:* The reference was added to line 323.

L348: What is the meaning of “evidence based monitoring of mitoPO2”?

*Answer:* We have rephrased line 348.

L353: It should be stated that this was intraoperatively. Other studies reported COMET-measurements in healthy controls (hemodynamically stable).

*Answer:* We have added ‘intraoperative’ to the sentence.

L355: Only neurosurgical patients should be added.

*Answer:* We have added ‘only measured in neurosurgical patients’ to the limitations.

***Additional topics, which could be addressed:***

Is the skin mitoPO2 a good surrogate parameter for the central or general oxygenation?

*Answer:* No, we do not think cutaneous mitoPO_2_ is a surrogate parameter for general oxygenation of the body. Our hypothesis is that, due to physiological compensation mechanisms, the skin is the first organ to suffer from detrimental physiological alterations (hypoxia, anemia, low cardiac output etc.) and therefore can serve as a ‘canary’ of the body. In our current thinking mitoPO2 will not be a replacement for e.g. hemoglobin saturation measurements. We hope to develop an early indicator of physiologic decompensation that is complimentary to existing clinical means of accessing oxygenation status.

Only neurosurgical patients. What other body area would be feasible e.g. for cardiac surgery?

*Answer:* Recently, our group has published a study in cardiothoracic surgery patients. In this study, measurements were performed on the upper arm. We believe that this is a good alternative, because it is also centrally located.

Would the authors expect different mitoPO2 values in different body regions?

*Answer:* In principle if all skin would have the same temperature, we would not expect a difference. However, as seen in this study, temperature has an effect of mitoPO_2_. Therefore, we expect the more distal regions, like hands and feet to have a different mitoPO_2_ value.

General Comment

I see two options for this manuscript:

I: If the primary aim of the manuscript is to show the feasibility and applicability of COMET-measurements during (neuro-) surgery, some of the reported results could be shortened (e.g. O2C measurements). In addition, more results on signal quality changes, excluded data

II: The manuscript could additionally focus on associative analyses. The great advantage of the design is the parallel assessment of mitoPO2, O2C variables and other covariates. Nonetheless, I’m aware that this would switch the focus of the current manuscript and would be very time consuming.
*Answer:* After careful consideration, we have chosen to perform the additional analysis and have added this to the manuscript. We want to thank the reviewer for this suggestion.

Reviewer #3:

Major Comments:

There is no discussion of the validity of the mitoPO2 monitor. What the face validity of this monitoring device is, in other words, if this device measures what it claims to measure, mitochondrial oxygenation, is not discussed. This discussion needs to include preclinical studies that are foundational for the development of this monitor. For example, are there animal studies performed to show the validity of this measurement? Was this measurement compared to other types of the mitoPO2 measurement or any existing related monitors? Content validity, whether this measurement covers all of the thing the authors are trying to measure, may deserve a consideration, too. If the device has not been tested for the validity, feasibility testing seems to be useless.
*Answer:* The PpIX-TLST has been in development from 2006 and has been subject to a number of reviews covering many of the above mentioned questions [2, 3]. We understand it is interesting for the PLOS ONE readers and have added a short paragraph on calibration and validation in the introduction.

A major scientific question is the role of mitoPO2. A possible role of mitoPO2 may be an early detection of tissue/cellular hypoxia before currently existing hemodynamic and oxygen monitors can do so. If that is the question, I believe that large animal studies, such as pigs, can answer the question in much more controlled conditions. Are these studies done in animals?
*Answer:* The reviewer is correct and the suggested possible role of mitoPO2 is exactly what we are aiming at. And yes, the possible role of mitoPO2 is subject of large animal studies. In the study by Römers et al. [4], the mitoPO_2_ decreased before the mean arterial pressure and serum lactate during a porcine hemodilution protocol. Follow up animal studies are under way, as our focus is on translational research. The lessons (and questions) learned in our ongoing clinical work will be subject of investigation in the laboratory.

Related to the above question, characteristics of the measurement needs to be explained. What is the temporal resolution of this measurement? Can the measurement reflect the mitoPO2 change at the second, minute or longer-level? How is the 5-min interval determined? The temporal resolution is important for interpreting the data and the usefulness as a monitor. In addition, how does the skin mitoPO2 reflect major organ mitoPO2? Are they correlated? If there are discrepancies between skin and other organs, the monitoring of the skin mitoPO2 may not be helpful. Further, the authors discuss that the decline of mitoPO2 during surgery may be associated with the skin temperature change. How the skin and ambient temperatures and warming methods affect the mitoPO2 should be easily tested on animals.
*Answer:* A single mitoPO_2_ measurement takes up to 6 milliseconds. The technical implementation in the COMET allows for one measurement every second, but in our laboratory setups we can measure up to 20 Hz (the limitation here is the laser repetition rate).

In light of the question of the correlation between skin mitoPO_2_ and other organs please see our answer to reviewer 2:
*Answer:* This is a valid and intriguing question. We have previously measured the mitoPO_2_ in different organs as well as the skin. The exact mitoPO_2_ values were differing between the locations, but the mitoPO_2_ showed the same behavior in all measurement sites after LPS administration [5]. We are currently investigating the canary function of the skin for other organs in a study with porcine models.’
To test the effect of warming methods on mitoPO_2_ is a good suggestion. We have not tested this as we were unaware of this effect. In future animal studies we will include the above mentioned suggestion as a research objective.

This study measured mitoPO_2_ in the limited time period (stable intraoperative) and in the limited patients. Anesthesia induction and emergence, hemodynamic instability, blood transfusion, moderate – high dose vasopressors are all excluded. Patients with presence of mitochondrial diseases, pregnancy or lactation are also excluded. However, the authors discuss a case report in which the mitoPO_2_ measurement was useful in a patient who had a large amount of blood loss. Therefore, it is not clear to this reviewer why the authors excluded all the instabilities, which seems to be a missed opportunity.
*Answer:* Please also see the answer to the question of reviewer 2: ‘The authors agree with the reviewer that measuring mitochondrial oxygenation in unstable hemodynamic conditions would be of interest. For example in patients with major blood loss. However, the primary aim of this study was to investigate the mitoPO_2_ in stable conditions, as it is the first time mitoPO_2_ was measured during surgery in adults. Seen as we do agree that unstable hemodynamic conditions are important as well, we are currently conducting measurements during major abdominal surgery in which major blood loss is expected. In a previous version, we had added two example cases from the previous mentioned population. However, these cases were removed after the editor’s comments. We have now added the two cases as a supplementary file.

Other Comments:

Are there any possible adverse effects of Alacare? Is Alacare absorbed systemically? Is Alacare approved for clinical use in the European nations?
*Answer:* Currently topical Alacare is approved for the treatment of actinic keratosis. Systemic Alacare is approved for visualisation of malignant gliomas during neurosurgery. After local application there is no clinically relevant systemic absorption. The possible adverse effects of Alacare include: itching, a burning feeling/pain, erythema, irritation, scab formation, exfoliation on the treated skin, hyper/hypopigmentation, blister formation, pustules, erosion, edema, bleeding of the treated skin and headache.

Line100: “Hypertension was medically induced during the hemostasis phase”. Is this induced hypertension for neurosurgical procedures?
*Answer:* Yes, in our hospital it is standard procedure to medically induce hypertension during the hemostasis phase for neurosurgical procedures.

Line 171: What are the causes of low signal quality?
*Answer:* A low signal quality is caused by a low signal to noise ratio of the optical signal. Potential reasons for this phenomenon include: interference of background light, pressure on the probe and insufficient uptake of aminolevulinic acid in the skin.

Line 198: When exactly the baseline mitoPO2 is measured? Is this before or after surgery started. Why is the awake mitoPO2 not included?
*Answer:* The patients are measured at the start of surgery after the induction of anaesthesia. This was due to two main factors, the first logistical and the second is the influence of ambient light. Thus only after the measurement site was covered from direct light sources by surgical covers could the measurement be conducted. Based on these experiences a sensor holder has been developed that covers the measurement site and enables the measurements in ambient lighting. In future studies it will therefore be possible to include peri- and postoperative measurements.

Line 276: The effect of anaesthesia should be tested in preclinical animal models.
*Answer:* Thank you for your suggestion. We are indeed considering to study the effects of anesthesia in a preclinical model. Also, we are currently setting up a clinical study in which we aim to analyze the effects of deep procedural sedation.

Figure 1 and 2: What are N20, N16, etc.? The averaged data across patients may not be helpful as all the surgical stimulation cannot be controlled. Are the data normalized or not normalized?
*Answer:* The N stands for the number of patients for the obtained means and SDs, we have added this to the figure legends.We appreciate the concern and have therefore added a mixed linear model which addresses the uncontrolled variables. Moreover, the data is not normalized.

Diagram for the mitoPO2 measurement mechanism may be helpful for the readers.
*Answer:* We have added an explanatory figure to the methods.

1.	Streng L, de Wijs CJ, Raat NJH, Specht PAC, Sneiders D, van der Kaaij M, et al. In Vivo and Ex Vivo Mitochondrial Function in COVID-19 Patients on the Intensive Care Unit. Biomedicines. 2022;10(7). Epub 2022/07/28. doi: 10.3390/biomedicines10071746. PubMed PMID: 35885051; PubMed Central PMCID: PMCPMC9313105.

2.	Mik EG. Special article: measuring mitochondrial oxygen tension: from basic principles to application in humans. Anesth Analg. 2013;117(4):834-46. Epub 2013/04/18. doi: 10.1213/ANE.0b013e31828f29da. PubMed PMID: 23592604.

3.	Mik EG, Balestra GM, Harms FA. Monitoring mitochondrial PO2: the next step. Current opinion in critical care. 2020;26(3):289-95. Epub 2020/04/30. doi: 10.1097/MCC.0000000000000719. PubMed PMID: 32348095.

4.	Römers LH, Bakker C, Dollée N, Hoeks SE, Lima A, Raat NJ, et al. Cutaneous Mitochondrial PO2, but Not Tissue Oxygen Saturation, Is an Early Indicator of the Physiologic Limit of Hemodilution in the Pig. Anesthesiology. 2016;125(1):124-32. PubMed PMID: 27176212.

5.	Harms FA, Bodmer SI, Raat NJ, Mik EG. Cutaneous mitochondrial respirometry: non-invasive monitoring of mitochondrial function. J Clin Monit Comput. 2015;29(4):509-19. Epub 2014/11/13. doi: 10.1007/s10877-014-9628-9. PubMed PMID: 25388510.
