# Supplementary material for: Monitoring of mitochondrial oxygen tension in the operating theatre: An observational study with the novel COMET® monitor
Source: PLoS One. 2023 Feb 9;18(2):e0278561. doi: 10.1371/journal.pone.0278561 (PMC9910761; doi:10.1371/journal.pone.0278561)
Supplement: Attachment — Submitted filename: Answers to the reviewer_1010.docx [file pone.0278561.s006.docx]

**Response to reviewers**

**Comments to the Author**

Reviewer #2: General comment
The authors have responded in detail to my questions and comments. The quality and informative value of the manuscript was significantly improved by the inclusion of the associative analyses. I have only a few minor comments and questions. From my point of view the manuscript is suitable for publication in PLOS ONE, when the remaining minor questions/comments are addressed.

Major
None

Minor
1. Line 84: The study referenced under [22] was performed in healthy controls.
he study performed in the ICU-setting is the current reference [40]:

Neu C, Baumbach P, Plooij AK, Skitek K, Götze J, von Loeffelholz C, Schmidt-Winter C, Coldewey SM. Non-invasive Assessment of Mitochondrial Oxygen Metabolism in the Critically Ill Patient Using the Protoporphyrin IX-Triplet State Lifetime Technique-A Feasibility Study. Front Immunol. 2020 May 7;11:757. doi: 10.3389/fimmu.2020.00757. PMID: 32457741; PMCID: PMC7221153.

*Answer:* The reference has been changed.


2. Line 164: please use lower case for „A self-adhesive“

*Answer:* The upper case A has been replaced with a lower case a.

3. Line 208: “correlation”
Please consider rephrasing to “association”
LMM is a form of regression analysis.

*Answer:* The sentence has been rephrased.

4. Line 255: “Correlation analysis using LMM”
Consider using the term “associative” or “regression” analysis. LMM is a form of regression analysis.

*Answer:* The sentence has been rephrased.

5. Line 386: „consequence of the changed parameters, the patient was resuscitation with red blood“
Please consider rephrasing (resuscitated?)

*Answer:* The sentence has been rephrased.

6. L343-344: “This implies that skin temperature is the cause 344 of the decrease in mitoPO2 after the start of the surgery.”
The current study design does not allow causal inferences. Please consider rephrasing (e. g. both were associated). The same holds for Lines 449-450.

*Answer:* The sentences have been rephrased.

7. Consider discussing the proportion of explained variance (marginal R²) for skin temperature. With 2.8 % the association is rather weak.

Answer: The following has been added to the discussion section:

‘In the mixed model for skin temperature, with subject as random effect and time point as both random and fixed effect, the marginal R^2^ was 3.4 %, which suggests that only a small part of the variation in mitoPO_2_ can be explained by skin temperature. When all the included covariates are considered, this marginal R_­_^2^ is 20.8% and the conditional R­^2^ 87.8 %. This insinuates that a large part of the variation in mitoPO_2_ is explained by the random effect part.’
 
Statistical analyses
8. Lines 199-201: “For a paired t-test, a sample size of 20 patients was calculated with an assumed mean difference of 12 mmHg and standard deviation of 18 mmHg, a type I error probability of 0.05 and a power of 80%.”

What was the rational of performing a sample size calculation for a paired t-test for a mainly descriptive study? Throughout the manuscript, no results of a paired test are reported. Was the initial aim to compare mitoPO2 at the beginning vs. the (individual) end of the surgery? This information or test might be added to the results part.

*Answer:* The following information has been added to the method section and results part:

‘A paired t-test was used to compare the mean baseline mitoPO_2_ value to the mean ‘end-of-surgery’ mitoPO_2_ value.’

‘When compared to the ‘end of surgery’ mitoPO_2_ (*M = 47, SD = 14)* using a paired t-test*,* mean baseline mitoPO_2_ (*M* = 60*, SD* = 19*)* was significantly higher (*t*(19) *=* 3.845*, p =* 0.001).’

Lines 210-211: In this model, the above-mentioned covariates were entered as fixed effects and subject number and time point were entered as random effects. What was the rational of adding a random intercept for „time point“? Please comment. Can you please provide the results if “time point” is neglected (just for the purpose of the review)?

*Answer:* A random effect for time point was added to look at the random slopes. We have tested the difference between a model with and without a random effect for time point (p < 0.001). Based on this, we have added the random effect for time point in the model. Furthermore, we added time point as a fixed effect as well.

The reviewer asked for the results if time point is neglected, which can be seen in the figure below.

**Figure 1.** Results of mixed linear model analysis without time point as random factor.

**Figure 2.** Results of mixed linear model analysis with time point as random factor.
