# Supplementary material for: Monitoring of mitochondrial oxygen tension in the operating theatre: An observational study with the novel COMET® monitor
Source: PLoS One. 2023 Feb 9;18(2):e0278561. doi: 10.1371/journal.pone.0278561 (PMC9910761; doi:10.1371/journal.pone.0278561)
Supplement: Attachment — Submitted filename: Answers to the reviewer.docx [file pone.0278561.s007.docx]

Answers to the reviewers concerning the manuscript ‘Monitoring of mitochondrial oxygen tension in the operating theatre: an observational study with the novel COMET® monitor.’

1. Reviewer #2: Maybe this is just a problem with the Word review mode: A: "with every increase of -1 degrees Celsius in skin temperature, mitoPO2 decreased by 1.76 mmHg"

*Answer: In our manuscript line 265 reads: With every increase of 1 degrees Celsius in skin temperature, mitoPO_2_ decreased by 1.76 mmHg’. Therefore, we believe that, as the reviewer suggested, this might be a problem with the Word review mode.*

2. B:"Additionally, the time point of the measurement, microvascular blood flow, StO2, and SpO2 were correlated with mitoPO2 (p < 0.001, p = 0.015, p = - 0.017 and p < 0.001". The bracket at the end of the sentence is missing-values cannot be less than 0.

*Answer: The bracket has been added. Furthermore, the misplaced minus sign before p = 0.017 has been removed.*

3. "It should be noted that due to the small sample size, the assumption that there is no measurement error has not been corrected for." Consider re-phrasing slightly.

*The sentence has been rephrased to: ‘It should be noted that measurement errors were not corrected for.’*
